# A multicenter whole-genome sequencing cohort of bleomycin-induced pneumonitis in predominantly clinically low-risk patients

**DOI:** 10.1007/s00438-026-02534-9

**Published:** 2026-10-30

**Authors:** Nadia Hitchen, Eleanor E. Handel, Claudia Leslie, Pietro R. Di Ciaccio, Anthony Joshua, Luke Ardolino, Peter Grimison, Florian Honeyball, Ben Tran, Linda McCarthy, Samuel Milliken, Nicholas Viiala, Shane Gangatharan, Ian Bilmon, Alison Y. Zhang, Jeremy Lewin, Garvin Marx, Caitlin Murphy, Anne Trinh, Nicola Wong, Leonard Goldstein, Sarah Kummerfeld, Matthew Hobbs, Megan B. Barnet

**Affiliations:** 1 Department of Medical Oncology, Peter MacCallum Cancer Centre, Melbourne, Australia; 2 Department of Oncology, The University of Melbourne, Melbourne, Australia; 3 The Kinghorn Cancer Centre, St Vincent’s Hospital, Darlinghurst, NSW Australia; 4 Sydney Adventist Hospital, Wahroonga, NSW Australia; 5 Chris O’Brien Lifehouse, Camperdown, NSW Australia; 6 Western Cancer Centre, Dubbo, NSW Australia; 7 Department of Haematology, Liverpool Hospital, Liverpool, NSW UK; 8 Fiona Stanley Hospital, Murdoch, WA Australia; 9 Macquarie University Hospital, Macquarie Park, NSW 10.Barwon Health, Geelong, Australia; 10 Barwon Health, Geelong, Australia; 11 Garvan Institute of Medical Research, Darlinghurst, NSW Australia; 12 School of Clinical Medicine, St Vincent’s Healthcare Clinical Campus, UNSW Medicine & Health, UNSW Sydney, Sydney, NSW Australia; 13 Australian and New Zealand Urogenital and Prostate Cancer Trials Group, Barangaroo, Australia; 14 The University of Sydney- School of Rural Health Dubbo, Dubbo, NSW Australia; 15 Deakin University, Victoria, Australia; 16 University of Western Australia, Crawley, UK; 17 School of Medicine and Psychology, Australian National University, Acton, ACT Australia

**Keywords:** Bleomycin-induced pneumonitis, Pharmacogenomics, Whole-genome sequencing, Pulmonary-toxicity

## Abstract

**Introduction:**

Bleomycin is a key component of curative-intent chemotherapy for germ cell tumours (GCT) and Hodgkin Lymphoma (HL), but its use is limited by bleomycin-induced pneumonitis (BIP). Although established clinical risk factors predict some cases, BIP also occurs without these. Preclinical models and small human studies implicate a contribution of drug metabolism, oxidative stress, immune and fibrotic pathways that may contribute to susceptibility. We aimed to identify germline genetic contributors to BIP in predominantly clinically low-risk patients to improve risk stratification in GCT and HL.

**Methods:**

We retrospectively identified 21 patients (13 GCT, 8 HL) who developed clinically significant BIP despite predominantly low clinical risk. Clinical data were collected and, where possible, blood samples were obtained for whole-genome sequencing (WGS). Analysis focused on a curated BIP-risk gene panel, derived from a literature review of genes/alleles implicated in BIP, alongside polygenic risk scores (PRS) for relevant lung and autoinflammatory/autoimmune pathology.

**Results:**

Median age was 39 (GCT) and 49 (HL) years; 67% were life-time non-smokers. Most patients with BIP experienced CTCAE grade < 3 (76%), and 52% received corticosteroids. WGS was performed in 18 individuals. Missense variants with low general population allele frequency were observed in 9 genes within the curated panel, although the significance of these remains unknown. PRS analysis in 12 individuals of European ancestry showed no association of BIP with established PRS for lung disease or autoinflammatory/autoimmune disease.

**Conclusion:**

Biologically plausible candidate germline variants involved in lung surfactant biology and inflammatory signaling were identified. These exploratory findings provide candidate genes and pathways for future investigations into the genetic basis of BIP.

**Supplementary Information:**

The online version contains supplementary material available at https://doi.org/10.1007/s00438-026-02534-9.

## Introduction

Bleomycin is a glycopeptide cytotoxic antibiotic originally isolated from *Streptomyces verticillus*. Its mechanism of action involves induction of DNA strand breaks, disruption of nucleic acid synthesis, and inhibition of cell division (Tamzali and Kemp-Symonds 2015). Clinically, bleomycin remains a cornerstone of curative-intent regimens for Hodgkin lymphoma (HL) and germ cell tumours (GCT) (Wit et al. 1997, Behringer et al. 2015). Bleomycin-induced pneumonitis (BIP) is the most feared complication, with a clinical spectrum ranging from subclinical decline in pulmonary function to progressive interstitial fibrosis and death. Reported rates vary by setting: in GCT, approximately 10% of patients may develop BIP, with a subset progressing to life-threatening disease, while in HL, similar or higher rates are observed despite lower cumulative doses (Simpson et al. 1998, Martin et al. 2005).

The pathogenesis of BIP is thought to involve oxidative stress within the pulmonary microenvironment, followed by immune-mediated alveolar injury and dysregulated repair leading to fibrosis (Burger et al. 1981). Experimental models have shown that overexpression of plasminogen activator inhibitor-1 (PAI-1) strongly correlates with hydroxyproline content and pulmonary fibrosis following inflammatory BIP (Eitzman et al. 1996, Kim et al. 2003). In addition, differential enzymatic inactivation plays a key role: bleomycin hydrolase (BLMH), the enzyme responsible for bleomycin detoxification, is expressed at relatively low levels in lung tissue compared with other organs (Jóna et al. 2016). This relative deficiency is thought to underlie the susceptibility of the lung to bleomycin-induced toxicity (Chen and Stubbe 2005). Genetic factors may further modify this risk; for example, a *BLMH* gene polymorphism (SNP A1450G) has been associated with variable enzyme activity and enhanced pulmonary toxicity, while carriers of H63D within *HFE*, a gene implicated in haemochromatosis, accumulate extracellular iron in lungs and have higher prevalence of BIP (Schoot et al. 2016). Preclinical and translational studies have implicated several biological pathways in BIP, including inflammatory signaling (IL6), fibrinolysis (SERPINE1/PAI-1), surfactant biology (SFTPA2, ABCA3), mucociliary clearance (MUC5B), telomere maintenance (TERT, RTEL1), antioxidant defense (*NFE2L2*/NRF2) and innate immune signaling (TLR4), with disruption of NRF2 and TLR2 pathways increasing susceptibility or severity of bleomycin-induced lung injury in animal models (Cho and Kleeberger 2007, Hancock et al. 2018).

Current clinical risk factors for BIP include older age, impaired renal function, and cumulative bleomycin dose (Simpson et al. 1998, O’Sullivan et al. 2003). Other potential clinical risk factors may include high flow oxygen exposure, pre-existing lung disease, smoking, high burden of pulmonary metastases, and concurrent use of granulocyte-colony stimulating factor with bleomycin (Roncolato et al. 2016). Among these, impaired renal function is considered the strongest predictor of BIP, as bleomycin is eliminated almost entirely through renal clearance (Reinert et al. 2013). Reduced glomerular filtration leads to higher systemic exposure, thereby substantially increasing the risk of pulmonary toxicity. This is particularly important for patients with high ventilatory demands (e.g., athletes, or those likely to undergo prolonged high-FiO₂ exposure peri-operatively), or in individuals with limited pulmonary reserve. Although there are clinical risk factors for BIP, toxicity remains unpredictable and can occur in those without recognized risk factors. In a large cohort of 835 patients with GCT, BIP occurred in 6.8% overall, importantly 2.5% of the patients had no identifiable clinical risk factors (O’Sullivan et al. 2003).

Emerging genomic data could refine risk stratification beyond traditional clinical parameters, enabling early identification of genetically predisposed patients for whom omission of bleomycin or substitution with alternative regimens (e.g., EP, VIP, or TIP in GCT; brentuximab-containing regimens in HL) could preserve cure rates while reducing late morbidity (Connors et al. 2018, Funt et al. 2021). In this exploratory study, we describe the clinical features of a national HL/GCT cohort who developed BIP despite predominantly lacking conventional risk factors, and apply whole-genome sequencing (WGS) to investigate candidate genes and polygenic architectures implicated in pulmonary injury and fibrosis. We hypothesised that predominantly clinically low-risk patients who develop BIP may harbor genetic predisposition to the toxicity. Whole genome sequencing of these individuals may identify single rare variants or common polygenic variation impacting bleomycin metabolism, pulmonary fibrosis or lung injury pathways.

## Methods

### Patient cohort

**Fig. 1:**
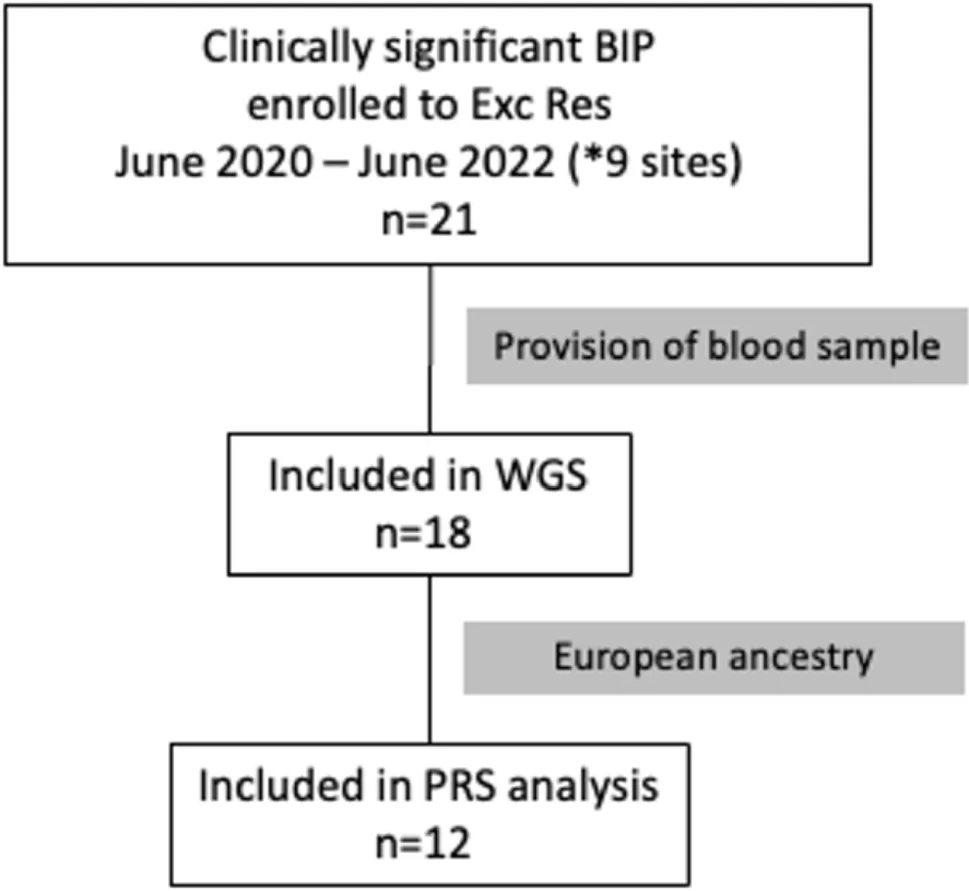
Consort Diagram. Of 21 patients with clinically significant BIP enrolled to the Exceptional Responders program between June 2020-June 2022 from 9 sites, 18 provided blood samples and were included in WGS analysis. Of these, 12 were of European ancestry and included in PRS analysis. BIP; bleomycin induced pneumonitis, WGS; whole genome sequencing, PRS; polygenic risk score

Patients with BIP following treatment for GCT or HL were retrospectively identified by clinicians from 9 treating institutions across Australia and enrolled into the Exceptional Responders program between June 2020 - June 2022 (Research). The study was promoted to clinicians through collaboration with the Australian and New Zealand Urogenital and Prostate (ANZUP) Cancer Trials Group for GCT and the Australasian Lymphoma Alliance (ALA) for HL. Eligible cases for the retrospective observational cohort study were selected as those with prior bleomycin exposure and clinically significant BIP, characterised by new respiratory symptoms with radiographic changes and pulmonary function test (PFT) decline, or asymptomatic decline in diffusing capacity of lungs for carbon monoxide (DLCO) > 25% during therapy not attributable to infection or tumour involvement during the study period (June 2020-June 2022). We excluded patients with high baseline clinical risk for BIP at treatment initiation, defined as estimated glomerular filtration rate (eGFR) ≤ 70 mL/min, cumulative bleomycin dose ≥ 400,000 IU, and age ≥ 70 years. The resulting cohort was therefore considered predominantly clinically low risk (Fig. 1). Following provision of informed consent, blood was drawn. Clinical data was collected retrospectively including demographics, smoking history, diagnosis, number of doses of bleomycin at BIP onset, cumulative bleomycin dose, clinical presentation of BIP (radiological or symptomatic), management (e.g. corticosteroids, immunosuppression), severity of BIP according to common terminology of adverse events (CTCAE V5.) and survival. The study was conducted under central ethics approval (HREC/18/SVH/171). This retrospective observational genomic study was conducted in accordance with the Helsinki Declaration of 1975 (revised in 2024) and is reported in accordance with STREGA guidelines(Little et al. 2009).

### Whole-genome sequencing

Whole-genome sequencing (WGS) was performed at the Garvan Institute of Medical Research. DNA was extracted from whole blood (Qiagen QIAamp DNA mini-Kit) and libraries were generated using the KAPA Hyper PCR-Free kit (Roche). 150-base paired-end reads were generated on an Illumina NovaSeq 6000 instrument, to a minimum 30x depth.

### Data processing and variant calling

Raw reads were aligned to the GRCh38 reference genome sequence using the BWA-MEM aligner software (version 0.7.15-r1140) and sorted and duplicate-marked with the program bamsormadup from the biobambam2 software project. Germline variant discovery was made by following the best practices guideline of GATK (Auwera Gvd, O’Connor 2020) using version 4.1.8.0. WGS data has been deposited with the European Genome-phenome Archive under accession number EGAD50000002221. Structural variants and copy number variants were called using the GermlineStructuralV-nf pipeline (Chukwu et al. 2024) and variants potentially affecting the panel of genes were inspected in the IGV genome browser (Robinson et al. 2011).

### Variant analysis

For variant filtering, variants were imported into an instance of the web-based tool seqr (Pais et al. 2022). Based on a review of the literature for genes/alleles implicated in BIP risk, a curated panel of 29 candidate genes (BIP panel) and associated regulatory regions implicated in bleomycin toxicity was compiled. This panel included ABCA3, BLMH, CSF3, CSF3R, CXCL1, CXCL2, CXCL8, DSP, IL17A, IL6, IL6ST, LOC110806263, LOC110806306, MUC5AC, MUC5B, NFE2L2, PARN, RTEL1, RTEL1-TNFRSF6B, SERPINE1, SFTPA1, SFTPA2, SFTPB, SFTPC, STN1, TERC, TERT, TLR4, and TNF (Supplementary File 1). Data was firstly filtered for location (used to retain only variants within our genes of interest), then genotype quality “high” (viz. pass variants only, minimum genotype quality 20, and minimum allele balance 25%). Subsequently, variants were filtered based on Ensembl Variant Effect Predictor impact (“moderate”, “high”) or ClinVar predicted pathogenicity (“pathogenic”, “likely pathogenic”). Allelic frequency (AF) filters were used to assess for enrichment of rare variants (population AF < 0.01), or relative depletion of common variants (population AF > 0.05). Expected allele frequencies were based on GnomAD v4.0 and p-values calculated using Fisher’s exact test. IGV was used to assess the reliability of the sequence alignments used to call candidate SNVs. Complex haplotypes were compared with sequences of haplotypes called by Locityper on 1000 Genomes project samples (https://locityper.vercel.app/available_data#locityper-genotypes-for-1kgp*)*.

Pharmacogenetic variant annotations of bleomycin collated from pharmacogenetic studies were retrieved from ClinPGx (formerly PharmGKB; https://www.clinpgx.org/chemical/PA448645). These included 10 variants for which a study has reported a significant association between a particular genotype and bleomycin toxicity (not specific to lung toxicity).

### Polygenic risk score analysis

Polygenic risk scores (PRS) were selected from the Polygenic Score Catalog based on their biological relevance to bleomycin-induced pneumonitis. Specifically, we included validated PRS for autoimmune and autoinflammatory diseases, reflecting the shared genetic architecture observed across immune-mediated conditions, together with pulmonary phenotypes characterised by immune dysregulation, fibrosis or malignancy. PRS analysis followed the approach of Chen et al.(Chen et al. 2024). European ancestry was defined by multi-dimensional scaling (MDS) of variant data projected onto reference populations from the 1000 Genomes Project. We compared PRS for European patients with BIP with a reference group of 667 European individuals from the 1000 Genomes Project (see https://pgsc-calc.readthedocs.io/en/latest/explanation/geneticancestry.html*).* Scores were calculated using the pgs_calc tool (Lambert et al. 2024) and applied to 38 PRS scoring files from the Polygenic Score Catalog (https://www.pgscatalog.org*)* (Lambert et al. 2024). The 38 catalog records are associated with lung function and were selected by querying the catalog with relevant phenotypic terms including “idiopathic pulmonary fibrosis”, “interstitial lung disease”, “lung function”, and “lung cancer” (Supplementary File 2). We also added several scores relevant to autoimmune diseases (Chen et al. 2024). We used Welch’s two-sample *t*-test to assess differences in Z_MostSimilarPop (from pgsc_calc) between our data and the reference group for all PRSs. To correct for multiple comparisons, we used a Bonferroni-adjusted alpha level of 0.0013 (0.05/38).

Given the rarity of both the underlying disease and bleomycin-induced pneumonitis, the study sample was determined by the number of eligible patients that could be identified rather than by a statistical power calculation. This exploratory study was designed to identify potentially relevant germline variants that may predispose to BIP. As the effects of individual variants are expected to be modest and genetically heterogeneous, substantially larger cohorts would be required to definitively establish causal genetic associations. Accordingly, findings from this study should be considered hypothesis-generating and intended to inform future validation in independent cohorts.

### Statistical analysis

Continuous variables were summarised using medians with ranges, as appropriate, and categorical variables were summarised using frequencies and percentages. Given the exploratory nature of the study and the sample size, statistical analyses were primarily descriptive.

For candidate variant analyses, observed allele frequencies in the study cohort were compared with reference population frequencies from the Genome Aggregation Database (gnomAD v4.0) using Fisher’s exact test. Rare variants were defined as those with a population allele frequency < 0.01 and common variants as those with allele frequency > 0.05.

For polygenic risk score analyses, differences in standardised PRS between the study cohort and the European reference population from the 1000 Genomes Project were assessed using Welch’s two-sample *t*-test. To account for multiple testing across the 38 polygenic risk scores evaluated, statistical significance was defined using a Bonferroni-adjusted threshold of *p* < 0.0013 (0.05/38). All statistical tests were two-sided, and p values < 0.05 were considered statistically significant unless otherwise specified for multiple comparison analyses.

## Results

### Patient cohort

A total of 21 patients were included in the descriptive analysis (Table 1). The cohort comprised 13 patients with GCT (62%) and 8 with HL (38%). The median age at diagnosis was 39 years for GCT and 49 years for HL. Most patients had no history of smoking (*n* = 14, 67%). Most BIP cases were CTCAE grade < 3 (*n* = 16, 76%). Two deaths occurred among patients exposed to bleomycin: one patient with HL treated with ABVD died in ICU from BIP complicated by acute infection, representing the single BIP-related death in the cohort, a second patient with GCT (treated at age 33 for seminoma/ teratoma) experienced grade 4 BIP and later died at age 54 from hypoxic respiratory failure due to COVID-19 in the context of bleomycin-related pulmonary fibrosis. Corticosteroid therapy was administered in 52% (*n* = 11) of cases, and 10% (*n* = 2) received additional immunosuppressive agents. Prior to BIP diagnosis, the median number of bleomycin doses was 9 in GCT and 4 in HL, with corresponding median cumulative doses of 270,000 IU and 69,200 IU, respectively. Of the 21 patients with BIP within the descriptive analysis, 18 provided blood for WGS and were assessed for single-nucleotide and structural variants (Fig. 1).

**Table 1:** Baseline Characteristics of BIP cohort

Characteristic	Hodgkin’s lymphoma (*n* = 8)	Germ cell tumours (*n* = 13)
Sex		
Female Male	2 (25%) 6 (75%)	0 (0%)
		13 (100%)
Median age in years (range)	49 (25–68)	39 (24–66)
Current or Former Smoker	3 (38%)	4 (31%)
Median no. bleomycin doses* Cumulative dose (IU)	4 69,200	9 270,000
BIP presentation		
Radiographic changes Symptomatic	6 (75%) 6 (75%)	9 (69%) 11 (85%)
BIP severity		
CTCAE < 3 CTCAE ≥ 3	5 (62%) 3 (38%)	11 (85%) 2 (15%)
BIP Treatment		
Corticosteroids Other immunosuppression	4 (50%) 1 (13%)	7 (54%) 1 (8%)

*Prior to BIP onset

### Genomic variants with predicted high impact on BIP-related genes

**Fig. 2:**
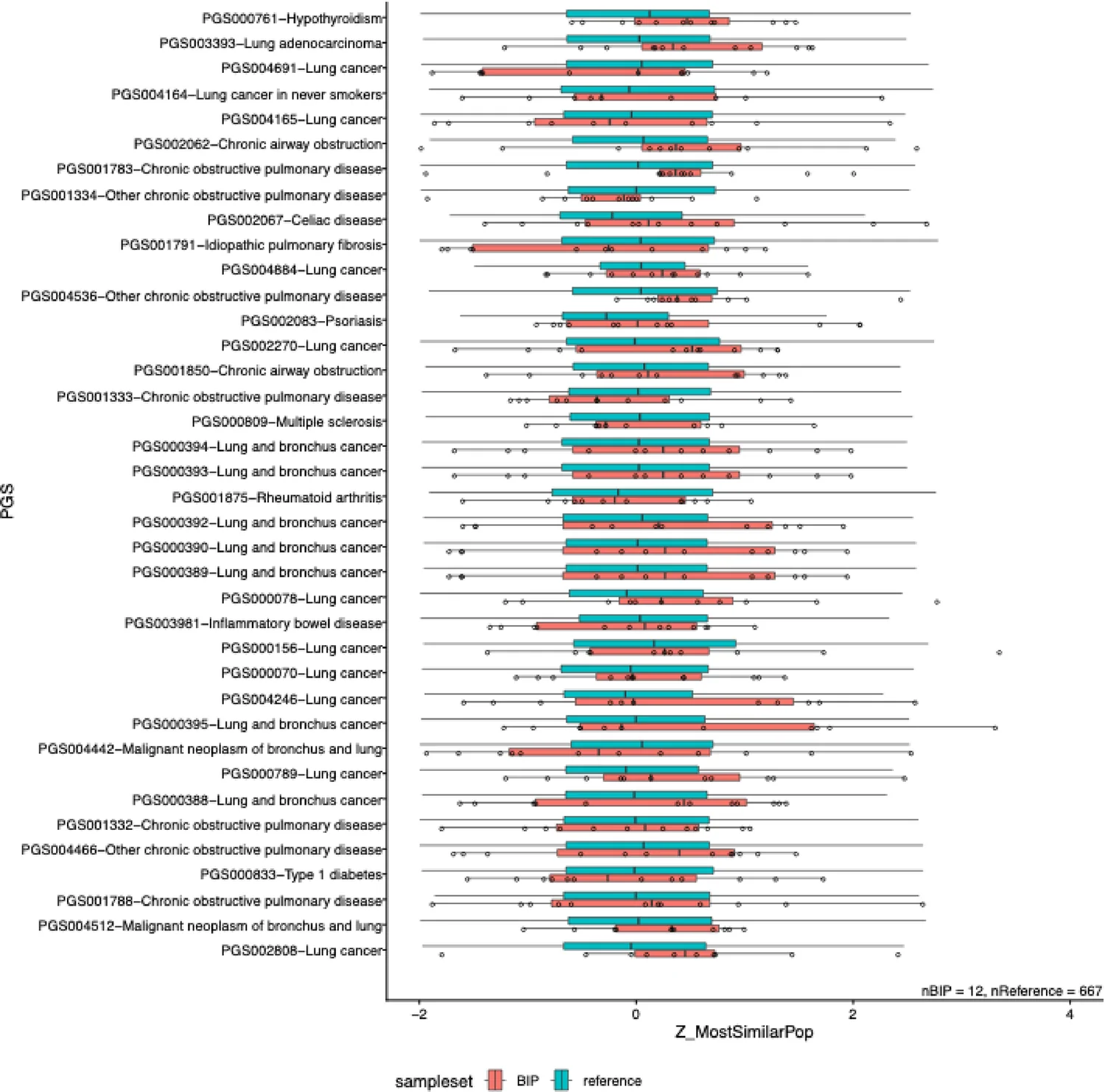
Boxplots showing the distribution of PRS scores in BIP group (*n* = 12) and reference group (*n* = 667) across 38 lung-related PRS items from the Polygenic Score Catalog (PGS). For the BIP group all individual points are shown

We applied several filtering strategies to obtain lists of candidate single nucleotide variants (SNVs) in our panel of BIP-related genes which might affect protein function, and subjected these candidates to further inspection. Firstly we looked for predicted high-impact variants; that is, variants likely to have a severe disruptive effect on protein expression and found 5 candidate variants in 4 genes (*MUC5AC* (2 variants), *NFE2L2*, *SFTPA2* and *PARN;* Supplementary File 3). We eliminated variants in *MUC5AC*,* NFE2L2* and *SFTPA2* from further consideration based on the following observations: (i) In *MUC5AC* two predicted frameshift variants (NC_000011.10:g.1185058_1185059del and NC_000011.10:g.1185062_1185063insAC) are located close together in a haplotype which is expected to be non-damaging because the 2 base-pair deletion and nearby 2 base-pair insertion maintain the MUC5AC reading frame. (ii) For both the *NFE2L2* variant (rs10179735; C > T) and *SFTPA2* variant (rs1975008; C > T) the GRCh38 reference allele is uncommon while the alt allele is the major allele, seen at high frequency in population datasets as well as in the BIP cohort where they are present in all samples. Because their AFs are so high we do not consider these variants to be of interest, and also note that the *SFTPA2* rs1975008 variant is classified as “Benign” in Clinvar. The remaining high-impact candidate variant rs12927927, a splice donor variant within *PARN*, the gene encoding telomere maintenance protein. We chose to disregard the variant because it affects only transcripts which are not protein-coding (ENST00000652541.1 and ENST00000563697.5). In summary, after curation there remain no high-impact variants potentially relevant to BIP (Fig. 2).

### SNVs with predicted moderate impact on BIP-related genes

We next looked at moderate-impact variants within genes of interest; that is, variants such as missense variants that may affect protein expression without causing complete loss of function. This resulted in 125 variants in 17 genes (Supplementary File 3). After applying an additional frequency filter (population AF ≤ 0.01) and checking sequence alignments in IGV there remained 14 rare variants in 9 genes. These 14 missense variants are distributed across 11 samples, and each is present in a single sample only. *MUC5AC* and *MUC5B*, which encode major secreted airway mucins and are characterized by highly repetitive, polymorphic genomic regions, showed the greatest sequence variability. In one patient, a distinctive ~ 20 kb region of high heterozygosity extended from the large central exon 31 to the 3’ end of *MUC5B*.

Two *ABCA3* variants, rs141621969 (Ala501Glu) and rs45592239 (Pro766Ser), were each observed in separate patients in our cohort and respectively have AFs 0.003 and 0.002 in the general population. Although neither has previously been linked to BIP, both alleles have been described in association with interstitial lung disease (Danhaive et al. 2008, Wambach et al. 2014). We also note that there is very little variation in any of our samples in a ~ 17 kb region of *ABCA3* covering exons 2–8; however, it appears that homozygosity in this region is common in the wider population.

Filtering instead for common population variants (allele frequency ≥ 0.05) with discordant low frequency within the BIP cohort showed relative depletion of two *IL6ST* variants which are in linkage disequilibrium (rs2228044 and rs2228043; Table 2) (Bonomi et al. 2020). The variants have allelic frequencies in the general population of 0.21 (rs2228044) and 0.17 (rs2228043) respectively, but are relatively deplete in the BIP cohort with one patient only carrying the haplotype (1 of 36 alleles for each; observed AF 0.028, p = < 0.01, not adjusted for multiple testing).

**Table 2:** Notable variants affecting genes of interest

Gene	SNP rs id	HGVS representation	Consequence	Genotypes	AF (BIP)	AF (GnomAD)^1^	Comments
BLMH	rs1050565	NC_000017.11:g.30249058T > C	missense variant (Ile443Val)	11 samples (ER136_BLEO3, ER141_BLEO6, ER151_BLEO10, ER152_BLEO11, ER163_BLEO13, ER164_BLEO14, ER165_BLEO15, ER174_BLEO17, ER176_BLEO19, ER181_BLEO22, ER188_BLEO26) are heterozygous (T/C) and 2 samples (ER140_BLEO5 and ER182_BLEO23) are homozygous (C/C)	0.42	0.29	ClinPGx reports that genotype C/C is associated with bleomycin toxicity and efficacy phenotypes
ABCA3	rs141621969	NC_000016.10:g.2300114G > T	missense variant (Ala501Glu)	One sample is heterozygous (G/T)	0.028	0.003	Possible enrichment in BIP cohort.
	rs45592239	NC_000016.10:g.2295708G > A	missense variant (Pro766Ser)	One sample is heterozygous (G/A)	0.028	0.002	Possible enrichment in BIP cohort.
IL6ST	rs2228044	NC_000005.10:g.55968325 C > G	missense variant (Gly148Arg)	One sample (ER136_BLEO3) is heterozygous (C/G).	0.028	0.21	Possible depletion in BIP cohort
	rs2228043	NC_000005.10:g.55956103G > C	missense variant (Leu397Val)	One sample (ER136_BLEO3) is heterozygous (G/C).	0.028	0.17	Possible depletion in BIP cohort

^1^Global (popmax) AF from GnomAD v4 genomes dataset

### Pharmacogenetic SNVs

Next, we referred to 10 alleles associated with all-cause bleomycin toxicity in the ClinPGx (formerly PharmGKB) pharmacogenetic database. All reported alleles were observed in our cohort (Supplementary File 3) which is as expected as all are relatively common alleles. The bleomycin hydrolase (*BLMH*) variant rs1050565 (Ile443Val) is of interest (Table 2) because ClinPGx annotations of this variant note that the C/C genotype is associated with bleomycin toxicity and efficacy phenotypes, and in particular reduced survival in bleomycin treatment of GCT (https://www.clinpgx.org/variantAnnotation/827807719). In our BIP cohort there are 2 homozygotes and 11 heterozygotes (Table 2). The C allele frequency is possibly enriched in BIP but this is not statistically significant (*p* = 0.09).

### SNVs in intergenic regions

Analysis of upstream regulatory regions of *TERT* and *TERC* (*LOC110806263* and *LOC110806306* respectively) returned 23 variants meeting quality requirements. After excluding low-quality and non-interpretable intronic changes (Gelfman et al. 2017), none of the variants were remarkable (Supplementary File 3).

### Structural variants

Finally, we examined predicted structural variants including indels, inversions and translocations, which are located within BIP panel genes, looking for any variants likely to impact gene expression. All were dismissed from further consideration because either they were (i) unlikely to have a damaging effect, such as small deletions affecting only intronic regions; or (ii) low-quality calls, typically with predicted breakpoints in repeat-rich regions of the genome where alignment of sequence reads was unreliable.

### Polygenic risk score (PRS) analysis

Because the majority of published PRS have been developed within patients of European ancestry, 12 patients matching this ancestry were considered for PRS analysis (Supplementary Fig. 1; Supplementary File 4). Patients were assigned a normative percentile rank based on their raw PRS relative to the reference population for each of 38 phenotypes, including pulmonary disorders and autoimmune/autoinflammatory diseases with known effects on systemic immune responses. After correction for multiple testing, there was no significant difference in distribution of PRS percentile ranks between BIP cases and the reference population.

## Discussion

In this multi-centre study, we describe the clinical and genomic features of BIP occurring in 21 patients despite predominantly low baseline clinical risk. The cohort reflected typical patterns of bleomycin use, with two-thirds of cases arising in GCT and one-third in HL, and median ages of 39 and 49 years, respectively. Notably, the median age at diagnosis for HL in this cohort was higher than the Australian population average. Nevertheless, despite predominantly low-risk clinical profile, nearly one quarter (24%) developed CTCAE grade ≥ 3 pneumonitis, and one patient died (HL), underscoring that BIP remains unpredictable and clinically significant. This prompted us to explore whether germline variation might contribute to susceptibility in this setting.

Initial filtering identified five variants with predicted high impact in four candidate genes, however, none remained potentially relevant following manual curation. Fourteen rare variants with predicted moderate impact were identified across nine genes of interest. Ten PharmGKB-annotated variants previously linked to bleomycin toxicity were also observed. We did not identify any single high-penetrance variants within a pre-defined set of genes implicated in BIP, or more broadly in pulmonary fibrosis, surfactant biology or inflammatory signaling across the cohort. We did observe potentially relevant enrichment or depletion in several variants that warrant validation in independent cohorts.

Variants in the mucin genes *MUC5AC* and *MUC5B* were identified. Accurate resolution of sequence changes within secreted mucin genes is challenging using short-read platforms due to large VNTR-rich central exons and so we inspected all sequence alignments in the IGV genome browser. Besides those missense variants given in Supplementary File 3 a distinct *MUC5B* haplotype spanning exon 31 in one patient is unlikely to be artefactual. The latter may warrant long-read validation, given the established role of *MUC5B* polymorphisms in mucin dysregulation leading to fibrotic lung disease (Hancock et al. 2018, Seibold et al. 2011).

Within *IL6ST*, the Gly148Arg - Leu397Val haplotype affects the extracellular domain of gp130 and has been associated with altered circulating soluble gp130 levels, potentially through effects on receptor conformation, stability or shedding, although its downstream effects on gp130–STAT3 signaling remain incompletely defined (Bonomi et al. 2020, Bonomi et al. 2020). Phosphorylated STAT3 is elevated in BIP, while experimental STAT3 inhibition mitigates bleomycin-induced lung injury (Pedroza et al. 2016), implicating dysregulated STAT3 signaling in BIP pathogenesis. The depletion of these variants in the BIP cohort (expected VAF 21% and 17% respectively; observed VAF 2.8%) did not withstand correction for multiple testing but, given the biological relevance of the gp130–STAT3 pathway, warrants independent validation. Common functional variants with modest effects in isolation may nevertheless influence susceptibility to inflammatory injury following immune stimulation. Furthermore, the frequencies of common alleles vary substantially across ancestral populations. For *IL6ST* Gly148Arg (rs2228044), for example, the minor (G) allele frequency within gnomAD ranges from 3.52% (East Asian) to 44.25% (African American); emphasising the broader implications of genomic diversity for treatment toxicity (Li et al. 2022, Balaji et al. 2023).

Rare variants were identified in *ABCA3*, a key regulator of surfactant metabolism in type II alveolar cells (Shulenin et al. 2004, Bullard and Nogee 2007, Nolan et al. 2025, Beers and Mulugeta 2017). Two of these variants (rs141621969 and rs45592239) have previously been reported in association with interstitial lung disease, providing rationale for further investigation of *ABCA3* in bleomycin-treated cohorts. Bleomycin hydrolase, an enzyme responsible for bleomycin inactivation, has been proposed as a potential modifier of bleomycin toxicity (Jóna et al. 2016). The rs1050565 (Ile443Val) variant has been hypothesised to alter protein function (Brömme et al. 1996) and was non-significantly enriched in our cohort, with variant allele frequency (VAF) of 0.41 (two homozygous and 11 heterozygous carriers) compared with expected VAF of 0.29. Although exploratory, this finding raises the possibility that inherited variation in bleomycin metabolism may contribute to interindividual susceptibility to bleomycin-induced toxicity. We also examined upstream regulatory regions of the telomerase genes *TERT* and *TERC*, given their role in interstitial lung disease (Arish et al. 2019, Borie et al. 2016) but found no variants of interest (neither SNVs nor SVs).

Polygenic risk scores (PRS) were evaluated in the subset of patients of European ancestry (*n* = 12). The available PRS were developed for common pulmonary and autoimmune/autoinflammatory traits rather than BIP, and PRS distributions within this small exploratory cohort were broad, limiting meaningful statistical comparisons. Although there is precedent for PRS developed for common diseases to predict related clinical phenotypes that share underlying biological mechanisms(Khan et al. 2021), the genetic architecture of BIP remains poorly understood. Accordingly, this negative finding should not be interpreted as evidence against a genetic contribution to BIP susceptibility. Rather, it highlights both the challenges of applying PRS derived from common genomic variation to a rare treatment toxicity with limited mechanistic insight, and the limitations of using reference cohorts that, while likely comprising predominantly individuals who would not develop BIP if exposed to bleomycin, cannot be phenotyped for susceptibility in the absence of drug exposure. These findings support the need for larger studies comparing bleomycin-exposed patients with and without BIP to define the inherited basis of susceptibility and enable the development of disease-specific PRS.

This study has several important limitations. The cohort was small and retrospectively assembled, reflecting the rarity of clinically significant BIP in predominantly low-risk patients, which consequently limited statistical power. The small number of affected individuals reduces the ability to robustly detect variant–phenotype associations, making it challenging to distinguish true driver mutations from random genomic variation and to identify potentially significant drivers with confidence given the absence of a control cohort without BIP. Polygenic analyses were further limited to individuals of European ancestry and therefore may not capture the genetic architecture of BIP across diverse populations. Despite these limitations, the national multi-centre design, systematic clinical annotation, and application of WGS represent key strengths of this study. The principal contribution is the establishment of a clinically annotated germline WGS resource for patients with BIP, providing a foundation for future collaborative studies. Taken together our findings are hypothesis-generating and broaden the landscape for future exploration. Susceptibility to BIP is likely determined by a complex interplay of risk-enhancing and risk-mitigating alleles, many of which vary substantially across ancestral groups and may contribute to differences in treatment toxicity. As bleomycin remains integral to curative regimens for many young patients, refining our understanding of these genetic modifiers is essential.

Larger, prospective real-world and clinical trial studies integrating genomics, functional analysis and detailed phenotyping will be required to define the biological determinants of BIP and other toxicities. Given that host susceptibility to bleomycin toxicity is poorly captured by current clinical stratification tools, large-scale genomic comparisons of affected and unaffected patients may help identify polygenic risk profiles reflecting convergent pathways in drug metabolism, inflammation and tissue repair, with particular focus in variants in genes such as *BLMH*, *ABCA3* and *IL6ST.* Improving our ability to anticipate bleomycin toxicity offers the opportunity to preserve its therapeutic benefit while minimising avoidable harm, advancing truly personalised treatment and improving long-term survivorship.

## Conclusion

In this exploratory study we did not identify a single unifying genomic signal to explain why BIP occurs in predominantly low risk patients, underscoring the complexity and likely multifactorial nature of this toxicity. Although several biologically plausible variants were observed within genes impacting surfactant biology and inflammatory signaling, these findings remain hypothesis generating and require validation. Global pooling of datasets is needed to increase statistical power and to this end we have deposited WGS data in the European Genome-Phenome Archive to facilitate collaboration.

## Supplementary Information

Below is the link to the electronic supplementary material.

Supplementary Material 1

Supplementary Material 2

Supplementary Material 3

Supplementary Material 4

Supplementary Material 5

## Data Availability

The sequencing data generated during this study, together with associated metadata, have been deposited in the European Genome-phenome Archive (EGA) under accession number (EGAD50000002221). Access to individual-level genomic data is managed through the EGA in accordance with participant consent and applicable ethical and privacy requirements.

## Abbreviations

GCT: Germ cell tumours
HL: Hodgkin Lymphoma
BIP: Bleomycin induced pneumonitis
WGS: Whole genome sequencing
PRS: Polygenic risk scores
BLMH: Bleomycin hydrolase
ANZUP: Australian and New Zealand Urogenital and Prostate (ANZUP) Cancer Trials Group
ALA: Australasian Lymphoma Alliance
PFT: Pulmonary function test
DLCO: Diffusing capacity of lungs for carbon monoxide
eGFR: Estimated glomerular filtration rate
CTCAE V5: Common terminology of adverse events
AF: Allelic frequency
MDS: Multi dimensional scaling
gnomAD v4.0: Genome Aggregation Database
SNVs: Single nucleotide variants
