# Supplementary material for: A multicenter whole-genome sequencing cohort of bleomycin-induced pneumonitis in predominantly clinically low-risk patients
Source: Mol Genet Genomics. 2026 Oct 30;301(1):208. doi: 10.1007/s00438-026-02534-9 (PMC13719838; doi:10.1007/s00438-026-02534-9)
Supplement: Supplementary file 1 [file 438_2026_2534_MOESM3_ESM.docx]

**Supplementary Figure 1**

Boxplots showing the distribution of PRS scores in BIP group (n = 12) and reference group (n = 667) across 38 lung-related PRS items from the Polygenic Score Catalog (PGS). For the BIP group all individual points are shown.
